# Age-related differences in appetitive trace conditioning and novel object recognition procedures

**DOI:** 10.1016/j.nlm.2019.107041

**Published:** 2019-10

**Authors:** Hayley J. Marshall, Marie A. Pezze, Kevin C.F. Fone, Helen J. Cassaday

**Affiliations:** University of Nottingham, Psychology, University Park, Nottingham NG72RD, United Kingdom

**Keywords:** Trace conditioning, Rat, Medial prefrontal cortex, Dorsal striatum, Nucleus accumbens, HPLC-ED

## Abstract

Appetitive trace conditioning (TC) was examined over 6 months in younger-adult (2–8 months) and middle-aged (12–18 months) male Wistar RccHan rats, to test for early age-related impairment in working memory. Novel object recognition (NOR) was included as a comparison task, to provide a positive control in the event that the expected impairment in TC was not demonstrated. The results showed that TC improved at both ages at the 2 s but not at the 10 s trace interval. There was, however, evidence for reduced improvement from one day to the next in the middle-aged cohort tested with the 2 s trace conditioned stimulus. Moreover, within the 10 s trace, responding progressively distributed later in the trace interval, in the younger-adult but not the middle-aged cohort. Middle-aged rats showed NOR discriminative impairment at a 24 h but not at a 10 min retention interval. Object exploration was overall reduced in middle-aged rats and further reduced longitudinally. At the end of the study, assessing neurochemistry by HPLC-ED showed reduced 5-HIAA/5-HT in the dorsal striatum of the middle-aged rats and some correlations between striatal 5-HIAA/5-HT and activity parameters. Overall the results suggest that, taken in isolation, age-related impairments may be overcome by experience. This recovery in performance was seen despite the drop in activity levels in older animals, which might be expected to contribute to cognitive decline.

## Highlights

• Longitudinal study of middle age in the rat with matched younger control cohort.
• Appetitive trace conditioning and novel object recognition tests of working memory.
• Transient between-groups working memory impairments aged 12 compared with 2 months.
• Object exploration reduced with age but working memory recovered.
• Object exploration and ITI nosepoking showed some correlation with 5-HIAA/5-HT.

## 1 Introduction

The purpose of the study was to test for early age-related impairment in working memory in a behavioural model of aging. Trace conditioning (TC) procedures are used to test animals’ ability to associate events across a time interval, in this case between a conditioned stimulus (CS, e.g. noise) offset and an unconditioned stimulus (US, e.g. food or footshock) onset (Kamin, 1965). Even quite short temporal discontiguity can be sufficient to reduce associative learning. Such learning impairment has been related to the cognitive demands of the TC procedure: to associate ‘what goes with what’ when events are separated in time relies on working memory, defined as the capacity to maintain ‘on line’ transitory information (Gilmartin et al., 2014, Goldman-Rakic, 1996, Sweatt, 2004). Thus, as a measure of working memory, TC provides a behavioural assay for age-related memory decline. Earlier studies have indeed shown that TC declines with normal aging in a number of species, including rabbits (Graves & Solomon, 1985), rats (McEchron et al., 2004, Moyer and Brown, 2006), mice (Galvez et al., 2011, Kishimoto et al., 2001), and man (Cheng et al., 2010, Knuttinen et al., 2001). TC impairment has also been reported in a mouse model of senescence (Lopez-Ramos, Jurado-Parras, Sanfeliu, Acuna-Castroviejo, & Delgado-Garcia, 2012).

TC is reliably demonstrated in both appetitive (e.g. food US) and aversive (e.g. footshock US) procedures. However, aversive procedures have more typically been used in studies of its neural substrates (Gilmartin et al., 2014, McEchron et al., 2000). Aversive procedures have similarly been adopted for studies of age-related decline in TC (Galvez et al., 2011, Graves and Solomon, 1985, Kishimoto et al., 2001, McEchron et al., 2004, Moyer and Brown, 2006). As might be expected given its importance for normal working memory function, the hippocampus is known to be susceptible to age-related decline (Bizon, Lee, & Gallagher, 2004). Extra-hippocampal structures such as the nucleus accumbens (NAc) and medial prefrontal cortex (mPFC) are also likely to modulate age-related decline (Dempster, 1992, Ito et al., 2008).

With respect to the likely neuropharmacological substrates of age-related changes, various working memory deficits have been attributed to dysfunction in mPFC dopamine (DA) pathways (Arnsten, 1998, Cai and Arnsten, 1997, Goldman-Rakic and Brown, 1981, Harada et al., 2002, Robbins and Arnsten, 2009). Moreover, at the appropriate dose, DA D_1_ receptor agonists have been found to restore working memory deficits in aged monkeys (Arnsten, 1998, Cai and Arnsten, 1997).

In addition to our earlier studies of the neural substrates of TC using an aversive fear conditioning procedure (Cassaday et al., 2005, Pezze et al., 2017) we devised an appetitive food motivated TC procedure to test the generality of the effects of amphetamine (Kantini, Norman, & Cassaday, 2004) and NAc lesions (Cassaday et al., 2005). This was further developed for use in a series of micro-infusion studies: we have recently shown mPFC DA D_1_ receptor modulation of appetitive TC (Pezze, Marshall, & Cassaday, 2015), as well as muscarinic receptor modulation in mPFC (Pezze et al., 2017) and hippocampus (Pezze, Marshall, & Cassaday, 2018). However, these intervention studies have to date been conducted on young adult rats and it has yet to be established whether appetitive TC shows sensitivity to age-related decline, as is known to be the case for aversive TC.

Thus, the present study examined appetitive TC in a repeated measures design over the course of normal aging in naïve rats, using appetitive procedures requiring many learning trials and hence suitable for the repeated testing. Based on our earlier experimental studies (Kantini et al., 2004, Pezze et al., 2015a, Pezze et al., 2017, Pezze et al., 2018), we hypothesised that older rats’ ability to condition over a 2 s trace interval would be reduced and that the timing of their responses within the longer trace interval might be impaired (normally rats come to distribute their responding towards the end of a 10 s trace). This was a longitudinal study in the sense that two groups of rats were examined over a 6 month timeframe; the developmental timepoints examined were from adulthood to early middle age vs from early to late middle age. Thus the design enabled us to test the further hypothesis that any differences seen between groups at the first point of comparison should also accentuate or emerge within groups as the cohorts aged.

The age range 12–18 months (i.e. early to late middle age) was selected because early behavioural changes are key to the detection of biomarkers for the onset of age-related cognitive decline, prior to the onset of irreversible neuropathological changes. Thus we set out to examine TC as a prospective behavioural screening tool, to assess the efficacy of treatments to restore cognitive function. Measures at different timepoints (every 6 weeks) enabled us to track age-related decline in the ability to condition a noise CS with food US over a trace interval. Importantly, a matched cohort of younger adult rats was conditioned under identical conditions but from 2 to 8 months of age, to provide some control for the effects of repeated training which would potentially offset age-related decline. Delay conditioning can (in principle) provide a suitable control, against which assess changes in TC. However, delay conditioning is itself susceptible to age-related decline (Cheng et al., 2010). In the present study, we therefore examined two different trace intervals in order to profile any age-related change at two levels of baseline performance using both a short (2 s) and a longer (10 s) trace interval.

Novel object recognition (NOR) was included as a comparison task, to provide a positive control in the event the expected impairment in TC was not demonstrated. The exploratory behaviours measured in NOR tasks are spontaneous, relying on rodents’ preference for (relatively) novel objects or locations, no further motivation is required (Ennaceur & Delacour, 1988). NOR tests can also be conducted repeatedly (using different sets of objects) and are thus suitable to examine age-related decline in object exploration and recognition memory using within-subjects designs (Lyon, Saksida, & Bussey, 2012). In rats, normal aging has been reported to impair recognition memory measured in the standard NOR object identity variant tested at long (24 h) but not short delays (Burke, Wallace, Nematollahi, Uprety, & Barnes, 2010) and aged rats’ NOR performance has been used to screen for cognitive enhancers (Lamirault & Simon, 2001). NOR based on object identity has been found to be impaired in a mouse model of sensescence (Lopez-Ramos et al., 2012) as well as in the scopolamine model of age-related cognitive decline (Hirst et al., 2006). Moreover, using analogous procedures (to examine NOR using 2D images rather than objects), recognition memory has been found to show age-related decline in non-demented elderly human participants, using both novel image identity and novel image location variants (Haley, Berteau-Pavy, Berteau-Pavy, & Raber, 2012). Thus, NOR has excellent independent validity as a behavioural model for aging. NOR tests are also sensitive to manipulation of both dopaminergic and serotonergic neurotransmission in the PFC (Lamirault and Simon, 2001, Lyon et al., 2012, Meffre et al., 2012, Watson et al., 2012).

After the final timepoint tests, tissue samples were collected from mPFC, dorsal and ventral striatum (NAc) for later assay by high-pressure liquid chromatography with electrochemical detection (HPLC-ED), in order to examine how age-related behavioural changes related to neuropharmacological substrates. The monoamine neurotransmitters and brain regions examined were selected because of their likely relevance to the cognitive changes anticipated and guided by our earlier experimental studies of TC (Cassaday et al., 2005, Kantini et al., 2004, Pezze et al., 2015a) as well by the effects of NAc (Nelson, Thur, Marsden, & Cassaday, 2010) and mPFC DA depletion (Nelson, Cooper, Thur, Marsden, & Cassaday, 2011) or D_1_ receptor modulation on NOR (Pezze, Marshall, Fone, & Cassaday, 2015). The hypothesis to be tested was that age-related decline in monoamine function would be apparent by late middle age. We also tested for correlations between neurochemical measures of monoamine function and behavioural indices related to activity as well as cognitive performance.

## 2 Materials and methods

### 2.1 Subjects

On arrival in the laboratory, male Wistar RccHan rats (Harlan) were caged in weight-matched groups of 4 in large double-decker individually ventilated cages (IVCs) which also provide animals with the opportunity to retreat from social interaction. They were kept on a 12:12 h light/dark cycle with free access to water, as well as unrestricted access to food during a 2 week acclimatization period. After 4 days’ acclimatization, all rats were handled daily for 1 week on arrival and at least twice weekly thereafter for the duration on the study. Weights were recorded every 2–3 days, more frequently in the case of any sudden weight loss (disproportionate to the individuals’ average body weight), or if daily husbandry checks indicated any signs of malaise.

The 24 rats to be used as the middle-aged group arrived at 16 weeks of age (at 324–452 g) from the Harlan Netherlands barrier. Following a total 2 week acclimatization period, this first cohort (now at 335–472 g) was food restricted to limit the development of adiposity which plays a major role in the development of inflammation and insulin resistance over the course of aging, such that the older rats did not rapidly exceed 500–600 g (as per veterinary instructions). Weight control was achieved by housing the rats in weight-matched groups (with subsequent adjustments to their housing as necessary) and tailoring the daily food ration. In addition to the sucrose pellets provided during conditioning, all rats received approximately 5 g per 100 g bodyweight food ration up to 20 g per day, this ration was adjusted as necessary to allow for healthy weight gain and to stabilise weights once these exceeded 400 g. The home cage ration was available until it was consumed, as the duration of food deprivation at the point of testing was not critical. Water was available *ab libitum*. Housing density was reduced to 2 rats per cage if the rats became too large to be comfortably accommodated at the original stocking density of 4/cage. The rats were closely monitored in house until they reached the age of 12 months (at 466–701 g), at which point the experimental phase of the study commenced. Two rats developed non-specific signs (including lethargy, partial piloerection, pallor of the extremities, irregular respiration, slow nose bleeds and possible seizures) prior to any conditioning, one later became prone to nose bleeds. In total 3 rats showing non-specific symptoms were humanely killed.

The middle-aged cohort was confirmed pathogen free. However, rodent parvovirus infection was later identified at Harlan Netherlands and they were therefore unable to supply matched rats to meet the experimental schedule. Harlan (now trading as Envigo) UK were able to supply Wistar RccHan rats, of matched genetic background and health status, as required 2 weeks prior to the scheduled start of the experimental phase. The 24 rats to be used in the younger-adult group arrived at 6 weeks of age from the Harlan UK barrier (at 151–180 g). The younger adult rats were acclimatized and handled, as for the middle-aged group, until they reached the age of 2 months (at 221–275 g), at which point the experimental phase of the study commenced.

All rats were introduced to sucrose reward pellets in their home cage over two days following the introduction of food restriction for experimental purposes. Regulated procedures were carried out in accordance with the principles of laboratory animal care, specifically the United Kingdom (UK) Animals Scientific Procedures Act 1986, Project Licence number PPL 40/3716, with the approval of the University of Nottingham Animal Welfare and Ethical Review Body (AWERB) committee and following the ARRIVE guidelines.

### 2.2 Trace conditioning apparatus

Experimental testing was conducted within a set of 4 fully automated ventilated conditioning chambers, adapted for appetitive conditioning. The food magazine (recessed in a side-wall of each of the chambers) was constantly illuminated for the duration of the pre-conditioning and conditioning sessions (in which food was available, as distinct from the extinction tests in which food was never available). Thus the magazine light helped the rats to find the food (following the signalled deliveries) but was not predictive like the CS. Access to the magazine was via a magazine flap. Nose-pokes were recorded by the breaking of the photo beam within the food magazine. The US was two 45 mg sucrose pellets (Formula F, Noyes Precision Food, New Hampshire, UK) dispensed into the magazine. The CS was a mixed frequency noise, presented via a loudspeaker in the roof of the chamber, set at 80 dB and of 5 s duration. An experimental background stimulus was provided by three wall mounted stimulus lights and the house light flashing on (0.5 s) and off (0.5 s), continuously for the duration of the conditioning session. This ‘envelope stimulus’ provided an additional measure of contextual conditioning, as per earlier studies of the neural substrates of trace conditioning conducted in the same laboratory using the same behavioural procedure (Pezze et al., 2015a, Pezze et al., 2017, Pezze et al., 2018).

### 2.3 Trace conditioning procedures

Allocation to TC groups was counterbalanced by box. The two cohorts of rats were also run in a counterbalanced sequence over two replications at each assessment timepoint. On each conditioning day there were 30 pairings of noise CS and food presented at a 2 s or 10 s trace interval. Conditioning was conducted on two consecutive days at each of the five timepoints.

#### 2.3.1 Pre-conditioning

There were two days of shaping to accustom rats to eating from the magazine. On the first day the rats were shaped in pairs, on the second and subsequent days of pre-conditioning they were placed individually in the conditioning chambers. On each of the first two days, rats had access to a pre-load of 15 reward pellets with an additional 15 rewards over 15 min to familiarise rats with the food deliveries. The tray flap door was propped open on days 1 and 2 but was closed on subsequent days so that the rats were required to nose-poke the door open to collect food. Then followed two days of baseline sessions, during which there were 30 unsignalled rewards over 60 min, delivered on a variable interval around 2 min, to habituate rats to the sounds produced by food delivery.

#### 2.3.2 Conditioning

Depending on the experimental group, the sucrose US was delivered 2 s or 10 s after CS offset (in the two different trace groups). Thirty signalled reward deliveries were presented on a variable interval, with the constraint that the inter-trial-interval (ITI) was always at least 1.5 times longer than the inter-stimulus-interval (ISI) length. Throughout acquisition, the background stimulus (flashing lights) was presented continuously. This continuous presentation also encompassed the 2 s or 10 s ISI, as applicable, which added to the overall duration of a 60 min session so that conditioning sessions were of either 61 or 65 min total duration.

The dependent variables to assess TC were the number of nose-pokes during the 5 s CS and during the 2 s or 10 s trace interval between CS and US (the ISI). This responding was compared with that seen in the 5 s after the delivery of the US in acquisition; and in the remainder of the session (the ITI, which excluded responding in the ISI). Latency to enter the food magazine was not recorded and there was no way to measure latency to consume the rewards with the automated set-up in use. However, no food was left (by either cohort) at the end of the conditioning sessions.

Because it was continuously presented, contextual conditioning to the flashing lights background could not be distinguished from (age-related changes in) ITI responding over the course of acquisition. We therefore examined responding to the experimental background, over and above any contextual conditioning to the cues provided by the experimental chambers, with a final set of extinction tests conducted 2 weeks after the fifth conditioning session. Following the test format of earlier studies, these used 30 5 s presentations of the flashing light stimulus over 30 min (Pezze et al., 2015a, Pezze et al., 2017, Pezze et al., 2018). The number of nose-pokes was recorded as above.

There were five timepoints of TC testing: starting from when the rats were aged 54 (the middle-aged cohort) or 10 weeks (the younger-adult cohort); at 6 weekly intervals (measured from the start of behavioural procedures at each timepoint); up to when the rats were aged 78 (the middle-aged cohort) or 34 weeks (the younger-adult cohort).

### 2.4 Novel object recognition apparatus

Testing was conducted in a 38 × 40 cm opaque plastic rectangular arena with 54 cm high walls. An overhead camera (JVC Everio GZ-EX515) was used to record the rats’ exploratory behaviour. The object stimuli were provided by duplicate copies of bottles and flasks made of glass, metal or plastic, and of varied shape, colour and size, and of weight too heavy to be displaced by the rat. We used the same pair of objects to test all the rats at timepoint 1. A different pair was used for the re-test at timepoint 5, matched for discriminability on the basis of previous data (Nelson et al., 2010, Nelson et al., 2011, Pezze et al., 2015b). The test box and objects were cleaned with an alcohol-based solution (20% v/v) before each trial to remove odor cues.

### 2.5 Novel object recognition procedures

NOR procedures were based on established protocols (Nelson et al., 2010, Nelson et al., 2011, Pezze et al., 2015b). Rats were placed individually into the arena for 1 h to allow habituation to the arena. Objects were introduced at the sample stage, which was conducted the following day following a further 3 min habituation. During the 5 min sampling period, rats were exposed to two identical objects (placed in opposite corners of the arena) and their exploration was recorded over 5 min. Rats were next tested for 3 min, either after a 10 min retention interval on the same day or 24 h later (after being returned to the home cage in the interim). At test they were presented with an object identical to that used at the sample stage and a novel object different from that used at the sample stage. The familiar object at test was an identical copy of the object seen at the sample stage. Object placements (left or right of arena) were counterbalanced. Time spent exploring each object was defined as directing the nose at the object at a distance of<1 cm and exploring it (i.e. sniffing or otherwise interacting with the object). Object exploration was not scored if the animal was in contact with but not facing the object, or if it sat on the object or used it as a prop (Ennaceur & Delacour, 1988). Time spent exploring the novel object divided by the total time spent exploring both objects during the choice phase was used to calculate a discrimination ratio, to take age-related differences in baseline exploration into account.

There were two timepoints of NOR testing: (1) two weeks after the first conditioning session when the rats were aged 56 (the middle-aged cohort) or 12 weeks (the younger-adult cohort); and (2) the week after the fifth and final conditioning session when the rats were aged 79 (the middle-aged cohort) or 35 weeks (the younger-adult cohort).

### 2.6 Neurochemical assay

Tissue samples were taken, using established micro-punch procedures (Nelson et al., 2010, Nelson et al., 2011). Following completion of the extinction tests, all rats were humanely killed, by concussion to the cranium and immediate dislocation of the neck followed by decapitation to remove the brains (which achieves exsanguination). This very rapid method of humane killing avoids the use of anaesthetic which would alter brain neurochemistry, enables collection of brain tissue regions prior to any enzymatic degradation of the monoamines, and is not associated with any brain tissue damage when done correctly. The method of killing the rats was approved by the University of Nottingham AWERB and conforms to a schedule 1 procedure under the UK Animals (Scientific Procedures) Act 1986.

The brains were removed rapidly and were placed dorsal side up in an ice-chilled rat brain matrix (Harvard Instruments, USA). Using ice-chilled razor blades, three 2 mm coronal brain sections were cut. The posterior side of the slices corresponded to approximately +3, +1 and −1 mm (anterior/posterior) from bregma according to the atlas of Paxinos and Watson (1998). The three 2 mm coronal sections were placed posterior side up onto an ice-chilled plate and tissue samples were taken using a 1.6 mm diameter stainless steel micro-punch: mPFC from the first section (+3 mm); NAc (ventral striatum) from the second section (+1 mm); and dorsal striatum from the third section (-1mm). The tissue punch samples were weighed, placed in 1.5 ml Eppendorf tubes, immediately frozen in liquid nitrogen and subsequently stored at −80 °C.

The tissue samples were later thawed, sonicated (Soniprep150: MSE Scientific Instruments) for 15 s in 250 µl (mPFC & NAc) or 500 µl (dorsal striatum) 0.1 M perchloric acid containing 0.4% sodium metabisulphite and 0.2% disodium ethylenediaminetetraacetic acid (EDTA), and centrifuged (17,400*g* for 20 min at 4 °C; Eppendorf centrifuge 5417R). The supernatant was then filtered through a 0.45 µM syringe tip filter (ThermoFisher Scientific). Neurotransmitter and metabolite levels in the samples were detected using HPLC-ED, separation was achieved using an autosampler (PerkinElmer Series 200), column (Targa C18 3 µm; 100x1.0 mm) and mobile phase, which was delivered at a flow rate of 45 µl/min by an isocratic pump (Dionex P680). Analytes were detected with a Decade SDC electrochemical detector fitted with a Sencell (Antec), using an oxidation potential of 0.75 V vs the Ag/AgCl reference at 32 °C. The mobile phase consisted of 20 mM potassium dihydrogen orthophosphate, 20 mM sodium acetate, 0.1 Mm EDTA, 0.15 mM octanesulfonic acid and 10% v/v methanol adjusted to pH 3.89 by adding concentrated orthophosphoric acid. Both samples and standard were prepared for injection at 10 µl. The external standard consisted of 5-HT, DA and their metabolites, in concentrations of 10^−7^, 4 × 10^−8^, 2 × 10^−8^ and 10^−8^ M for calibration.

The HPLC results were acquired using the Clarity Software data system. The protocol was designed to enable the monoamines and their major metabolites to be separated and quantified. No other metabolites or monoamines could be separated by this method. Moreover, the tissue samples were small, so to avoid the margin of error in the correction for tissue weight in the statistical analyses (see below), ratios to index 5-HT and DA utilisation were computed for each brain region from the raw levels of metabolite and neurotransmitter (i.e. 5-HIAA/5-HT and DOPAC/DA). HVA levels were not reliably determined in NAc and mPFC but [DOPAC + HVA]/DA could be examined in dorsal striatum.

### 2.7 Design and analysis

The dependent variable used to examine age-related differences in TC was the number of responses (nose-pokes into the food magazine) made during the CS presentations. To separate out effects on motor responding or motivation for food reward, similar analyses were conducted on the ITI and US response measures. Difference scores (CS minus the average ITI responding/5s) provided an adjusted measure of CS responding. Additionally, the responding of the animals during the 10 s trace interval between CS offset and US delivery was also examined for any age-related change in the distribution of responding within the trace interval. Thus, the 10 s ISI was broken down into two second bins of time and analysed using repeated measures ANOVAs by bins and days. Responding to the light background was examined only upon completion of the conditioning procedures at timepoint 5. Responding to 5 s light presentations was compared to that seen 5 s prior to the presentations (pre-stimulus) and 5 s after offset (post-stimulus), as well as in the ITI.

The behavioural data were analysed in mixed design with the between subjects factors of trace (at 2 levels: 2 s and 10 s) and cohort (at two levels: younger-adult and middle-aged). To assess effects over the course of acquisition, the repeated measures (within subjects) factors were timepoint (at 5 levels: 5 conditioning sessions at 6 weekly intervals) and days (at 2 levels: 2 days’ conditioning per timepoint). Additionally, within day acquisition was examined by blocks (at 6 levels: 6 blocks of 5 trials per day); and the distribution of responding within the 10 s trace interval was examined by bins (at 5 levels: 5 bins of 2 s within the 10 s trace). Responding to the light background was examined using the same factorial design applied to test within day acquisition to the noise CS, in this case in relation to six blocks of five unreinforced stimulus presentations (since there was only one day of extinction testing). Trend analyses of interactions involving timepoint, blocks or bins obviate the need for multiple comparisons (Howell, 2002). Selected comparisons, to identify in which (if any) bins the difference between the first and final timepoints were individually significant, were by paired comparisons *t-*test.

The dependent variable used to examine NOR was exploration time for novel vs familiar objects over the 3 min test presentation. Exploration times were compared in a mixed design with the between subjects factor of retention interval (at 2 levels: 10 min and 24 h) and cohort (at two levels: younger-adult and middle-aged); the within-subjects factors were familiarity (at 2 levels: novel versus familiar) to assess NOR and timepoint (at 2 levels: 1 and 5) to assess effects longitudinally over the course of aging (within the timeframe of the study). Further analyses of the discrimination ratios used a single between subjects factor of group (Abelson, 1995, Shaffer, 1977). Planned comparisons, to compare discriminative performance to the 0.5 value of the ratio which reflects chance level performance, were by one-tailed Student’s *t-*test.

Differences in neurotransmitter turnover between the cohorts at the final timepoint were determined by Student’s *t*-test. Additionally, associations between neurochemical and behavioural parameters were explored by correlational analyses. These analyses were conducted using summary scores that reflected TC and NOR performance (for both cohorts) at the final time point. For TC, we used CS, ISI, US and ITI responding at the final (fifth) timepoint (averaged over the two days). For NOR, we used sample exploration, and exploration of the novel versus familiar object at timepoint 5 (when the second NOR test was conducted).

## 3 Results

### 3.1 Pre-conditioning

The middle-aged group (M = 138.729 ± 16.703) responded overall less than the younger-adult cohort (M = 224.396, ±15.926), F(1, 42) = 13.778, p = 0.001. There were no significant effects by trace condition-to be, Fs < 1.

### 3.2 ITI responding

**Fig. 1:**
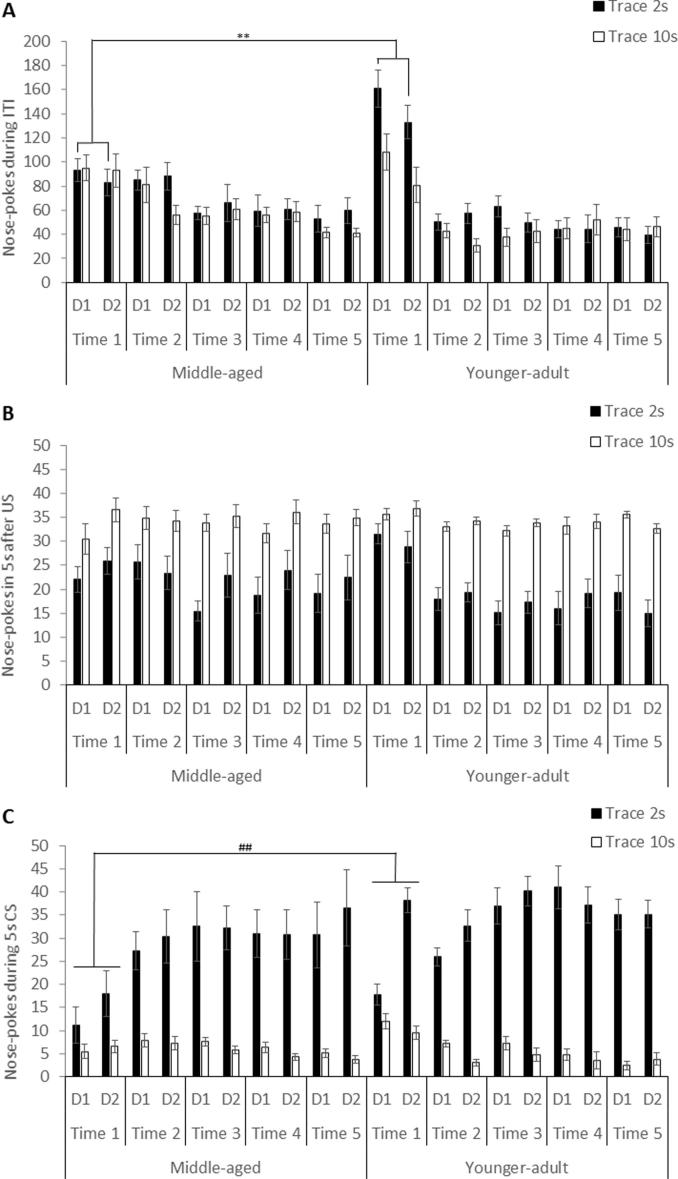
Mean nose-poke responding (±sem) during (A) the inter-trial-interval (ITI), (B) the 5 s after food unconditioned stimulus (US) delivery, or (C) the 5 s presentations of the conditioned stimulus (CS) in middle-aged and younger-adult cohorts of Wistar RccHan rats N = 9–12/cell. Asterisks indicate the significant difference between the age groups at timepoint 1 in the 2 s trace group, averaged across days, **p < 0.01). Hashtags indicate the significant difference between the age groups at timepoint 1, averaged across days and trace intervals, ^##^p < 0.01).

There was a significant effect of cohort × timepoint, F(4, 164) = 13.396, p < 0.001, as well as cohort × timepoint × trace, F(4, 164) = 5.561, p < 0.001. Timepoint was also significant by trace, F(4, 164) = 2.589, p = 0.039, as well as by day, F(4, 164) = 2.691, p = 0.033. The difference between the cohorts is of most importance because differences in ITI responding potentially confound the measure of TC provided by CS responding. The younger-adult cohort responded more in the ITI at timepoint 1, in the 2 s, t(20) = 3.642, p = 0.002, but not in the 10 s trace conditioned group, t(22) = 0.037, p = 0.971 (Fig. 1A).

### 3.3 US responding

In order to ascertain whether aged rats were less motivated by the sucrose rewards, responding in the 5 s following US delivery was also examined (in exactly the same way we measured responding during the 5 s CS presentations, reported below).

As might be expected, ANOVA showed a number of effects involving timepoint, including by cohort, F(4, 164) = 4.710, p = 0.001. This arose because whilst older rats maintained consistent levels of responding (Time 1 M = 28.528 ± 1.653; Time 5 M = 27.563 ± 1.893), the younger-adult rats showed some overall reduction in US responding as training progressed (Time 1 M = 31.938 ± 1.530; Time 5 M = 24.875 ± 1.753). Fig. 1B shows the same US data by day and trace for direct comparison with the CS and ITI data. Additionally, an effect of timepoint × trace, F(4, 164) = 4.178, p = 0.003, arose because responding overall reduced as conditioning progressed, in the 2 s but not the 10 s trace conditioned rats (Time 1 2 s M = 25.611 ± 1.653; Time 5 M = 18.979 ± 1.893; Time 1 10 s M = 34.854 ± 1.530; Time 5 10 s M = 33.458 ± 1.753). As can be seen in Fig. 1B, the three-way interaction of timepoint by cohort and day, F(4, 164) = 4.104, p = 0.003, did not relate to any systematic pattern in the data and will not be further discussed.

### 3.4 CS responding

This was assessed by the use of a difference measure, to adjust for ITI responding (described above). There was a significant effect of cohort × timepoint, F(4, 164) = 3.495, p = 0.009. As shown in Fig. 1C, older rats showed an overall lower level of nose-poking at timepoint 1, t(44) = 3.102. p = 0.003. However, there was no difference between the cohorts by timepoint 2, t(44) = 0.005, p = 0.996, and nose-poking in the older rats remained equivalent to that seen in the younger rats for the full duration of testing, at timepoint 5, t(43) = 0.381, p = 0.705.

There was also a timepoint × trace interaction, F(4, 164) = 13.673, p < 0.001, significant also in the linear trend, F(1, 41) = 19.066, p < 0.001. This arose because overall conditioning improved in both cohorts as the study progressed, at the 2 s but not at the 10 s trace interval. The three-way interactions with cohort were not significant. The four-way interaction, F(4, 164) = 3.654, p = 0.007, significant also in the linear trend, F(1, 41) = 11.422, p = 0.002, appeared to relate to older rats’ reduced improvement from one day to the next, at the early timepoint measures of CS responding at the 2 s trace interval (which is further examined below). ANOVA of the raw scores showed the same statistical outcomes and the raw scores are shown in Fig. 1C.

**Fig. 2:**
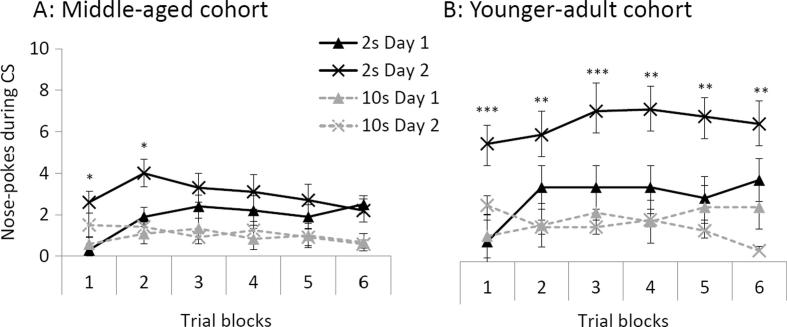
Mean nose-pokes during CS presentations are shown as a function of the 6 blocks of trials conducted on each of two days at timepoint 1 for (A) the middle-aged cohort and (B) the younger-adult cohort. Solid black lines denote rats conditioned at the 2 s trace interval and broken grey lines denote rats conditioned at the 10 s trace interval. Triangles denote day 1 conditioning and X symbols denote day 2 conditioning. Error bars represent the standard error of the mean. Asterisks indicate a significant difference between day 1 and day 2 responding for the 2 s trace conditioned groups, *p < 0.05, **p < 0.01, ***p < 0.001.

Initial acquisition was further examined by ANOVA of the timepoint 1 data (across the 6 blocks of trials for each of the 2 days of conditioning). Fig. 2 shows that the younger animals performed better on the 2-s appetitive TC task than the middle-aged group. The acquisition data are shown by 6 blocks of 5 trials (as there were in total 30 conditioning trials per session) for each of the two successive training days conducted at timepoint 1. Neither age group performed well in the 10-s trace variant, on either of the training days conducted at timepoint 1. However, acquisition was shown at the 2-s trace interval and with a marked improvement from the first to the second day in the younger cohort.

As would be expected based on the above analysis of CS responding averaged over the day, there were interactions between cohort and days, F(1, 42) = 4.525, p = 0.039 and cohort × trace × days, F(1, 42) = 12.601, p = 0.001. There was also a main effect of cohort, F(1, 42) = 15.678, p < 0.001, because the older rats responded overall less. Blocks was significant by trace, F(5, 210) = 4.236, p = 0.001, also in the linear trend, F(1, 42) = 8.085, p = 0.007, reflecting more rapid acquisition to the CS conditioned at 2 s versus 10 s. Of the interactions involving cohort and blocks none was significant, except for the four-way in the linear trend F(1, 42) = 6.956, p = 0.012, which was further explored by the selected comparisons shown in Fig. 2. ITI data was not recorded block-by-block so there were no corresponding difference scores for the analyses by blocks. However, the same statistical outcomes were confirmed when the timepoint 1 ITI responding was taken into account by ANCOVA.

### 3.5 ISI responding

**Fig. 3:**
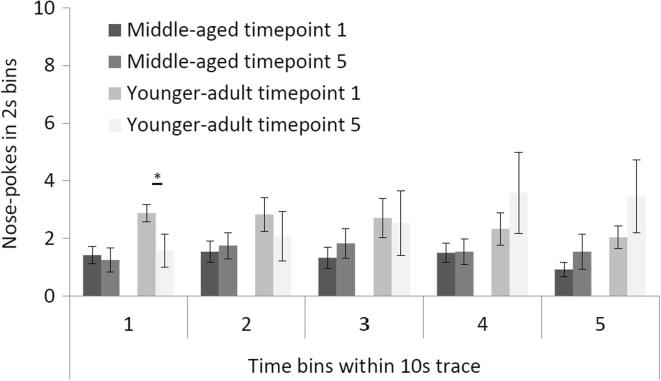
Mean nose-pokes during the five 2 s bins of the 10 s trace interval for the middle-aged cohort (darker greys) and the younger-adult cohort (lighter greys) and timepoint 1 (in each case a relatively darker shade) versus timepoint 5 (in each case a relatively lighter shade). Error bars represent the standard error of the mean. Asterisk indicates a significant difference between the timepoints (the younger-adult cohort bin 1), *p < 0.05.

There was an effect of cohort × bin, F(4, 88) = 2.628, p = 0.040, significant also in the linear trend, F(1, 22) = 4.450, p = 0.046, as well as a three-way interaction of cohort × bin × timepoint, F(16, 352) = 1.735, p = 0.039, significant also in the linear trend, F(1, 22) = 4.308, p = 0.05. Therefore the interactions arise because a linear relationship best describes the change in responding across the 2 s time bins of the 10 s trace and this is different by cohort and timepoint. Fig. 3 shows that the difference between the cohorts took the form that the younger-adult rats showed relatively increased nose-pokes for food towards the end of the 10 s trace interval at timepoint 5. The summary (Fig. 3) comparisons between timepoint 1 and timepoint 5 show that the younger adult rats shifted their responding toward the end of the trace interval by timepoint 5. The only individually significant difference was the drop in responding in the first 2 s bin, t(11) = 2.356, p = 0.038, but the direction of the difference between timepoint 1 and timepoint 5 was reversed in bin 5 (as reflected in the three-way interaction between cohort, bin and timepoint). Fig. 3 also shows that (as expected) anticipatory responding was overall low during the 10 s trace interval: total nose-pokes over the 10 s trace M = 10.038 (±1.759); total nose-pokes within the 2 s shorter trace (data not shown in Fig. 3), M = 18.821 (±1.900).

A further three-way interaction cohort × timepoint × day, F(4, 88) = 2.730, p = 0.034, did not relate to any systematic shift across the timepoints and will not be further discussed.

### 3.6 Light background

There were no effects of cohort on any of the corresponding measures of responding to the experimental background provided by the light stimulus (5 s pre-stimulus, 5 s stimulus, 5 s post-stimulus or in the ITI), maximum F(1, 41) = 1.234, p = 0.273. ANOVA of responding to successive presentations of the light over the course of extinction (by the 6 blocks of 5 presentations) confirmed this conclusion: there were no effects by cohort, maximum F(1, 41) = 1.234, p = 0.273. As would be expected, overall response levels within these sessions were relatively low: middle-aged cohort M = 19.708 (±4.391); younger-adult cohort M = 23.958 (±4.066).

### 3.7 Novel object recognition

There was a main effect of cohort because older rats explored overall less at the test stage, F(1, 41) = 30.053, p < 0.001. There was also an effect of timepoint, F(1, 41) = 15.120, p < 0.001, because rats explored overall less at timepoint 5. An interaction between timepoint and delay, F(1, 41) = 8.311, p = 0.006, arose because test exploration showed a greater drop at the 24 h retention interval.

The preference for the novel object was confirmed by the main effect of familiarity, F(1, 41) = 78.396, p < 0.001, which – as would be expected – depended on the retention delay, F(1, 41) = 11.729, p = 0.001, and the minute of the test session, F(2, 82) = 11.129, p < 0.001. Importantly, there was an interaction between familiarity and cohort, F(1, 41) = 4.129, p = 0.049, because the younger-adult cohort showed a greater difference in the exploration of the novel compared to the familiar object. However, none of the other interactions between familiarity, cohort and timepoint was significant, maximum F(2, 82) = 1.604, p = 0.207.

**Fig. 4:**
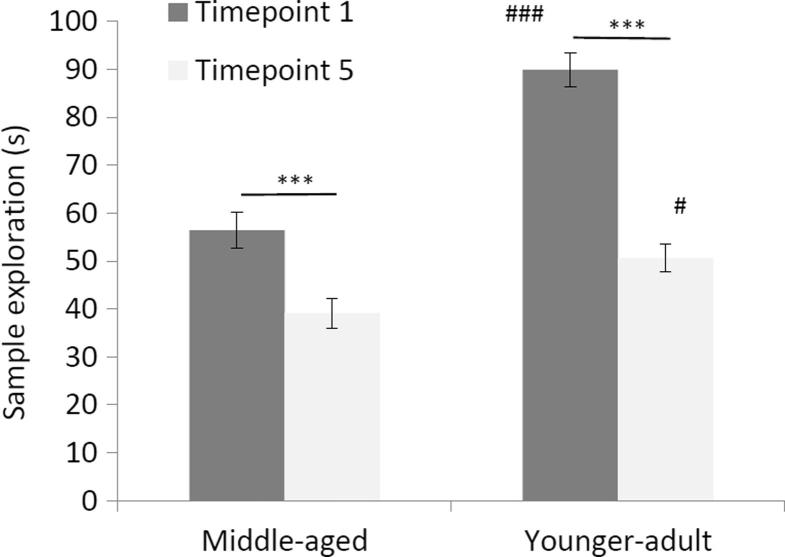
Total sample stage object exploration (s) at each of the two NOR test timepoints. Tests at timepoint 1 (dark grey bars) were conducted at 56 (the middle-aged cohort) and 12 weeks (the younger-adult cohort). Tests at timepoint 5 (light grey bars) were conducted at 79 (the middle aged cohort) and 35 weeks (the younger-adult cohort). Error bars represent the standard error of the mean. Asterisks show significant differences between timepoints, ***p < 0.001. Hashtags show significant differences between age groups at the corresponding timepoints, ^#^p < 0.05, ^###^p < 0.001.

As might be expected given the age-related effects on test exploration, there was also a clear effect of cohort on sample exploration, F(1, 41) = 34.171, p < 0.001, because the younger-adult group explored more than the older group. There were also effects of timepoint, F(1, 41) = 106.676, p < 0.001, as well as a timepoint by cohort interaction, F(1, 41) = 16.079, p < 0.001, because sample exploration reduced from timepoint 1 to timepoint 5, particularly in the younger-adult cohort which showed higher levels of sample exploration to begin with. Fig. 4 shows relatively impaired sample exploration in the middle-aged cohort, particularly at timepoint 1. However, the relatively higher levels of exploration seen in the younger cohort were not sustained. By the time they had reached 8 months of age (at timepoint 5) their levels of exploration were very similar to those seen in the middle-aged group to begin with (when they were 12 months of age), albeit still significantly higher than levels of exploration in the middle-aged group aged to 18 months.

**Fig. 5:**
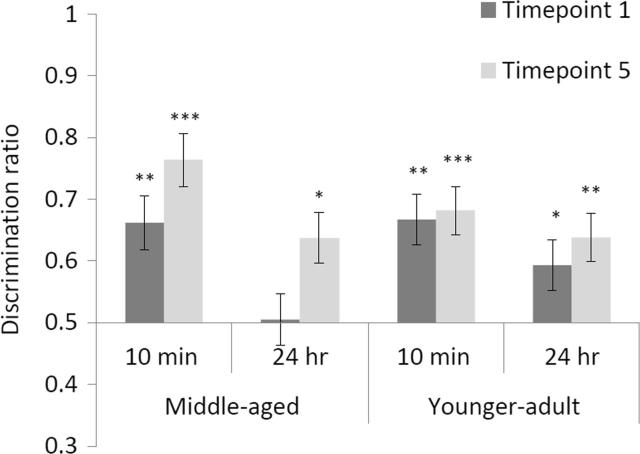
Discrimination ratio scores at each of the two NOR test timepoints. A ratio of 0.5 indicates no discrimination; scores above 0.5 indicate a preference for the novel object. Tests at timepoint 1 (dark grey bars) were conducted at 56 (the middle-aged cohort) and 12 weeks (the younger-adult cohort). Tests at timepoint 5 (light grey bars) were conducted at 79 (the middle-aged cohort) and 35 weeks (the younger-adult cohort). Error bars represent the standard error of the mean. Asterisks show performance significantly above the ratio 0.5, *p < 0.05, **p < 0.01, ***p < 0.001.

Discrimination ratios take differences in baseline exploration into account and simplify the analyses because the familiarity factor is removed. ANOVA of the discrimination ratios showed an effect of timepoint, F(1, 41) = 7.010, p = 0.011. However, this arose because of overall *improved* NOR performance at the second NOR test conducted at timepoint 5. As expected, there was an effect of delay, F(1, 41) = 10.633, p = 0.002 because NOR performance was better at the 10 min than at the 24 h retention interval. None of the interactions involving age was significant, maximum F(1, 41) = 2.449, p = 0.125. However, when the between subjects factors of cohort and delay were collapsed (Abelson, 1995, Shaffer, 1977), there was an overall effect of group at the first, F(3, 42) = 3.260, p = 0.031, but not at the final timepoint, F(3, 41) = 1.985, p = 0.131. This arose because, at the first test timepoint, older rats were at chance at the 24 h retention interval, t(10) = 0.124, p = 0.904, whereas the older rats allocated to the 10 min retention interval performed above chance, t(10) = 3.317, p = 0.008. Younger-adult groups were above chance at both timepoint 1 retention intervals, minimum t(11) = 2.336, p = 0.039. Counter to expectation, at the second NOR test (timepoint 5) all rats performed above chance, minimum t(10) = 2.816, p = 0.018. Fig. 5 shows that the middle-aged group showed improvement when re-tested at the final timepoint, from a lower baseline with the 24 h retention interval at the first NOR testing session.

### 3.8 Neurochemistry

**Fig. 6:**
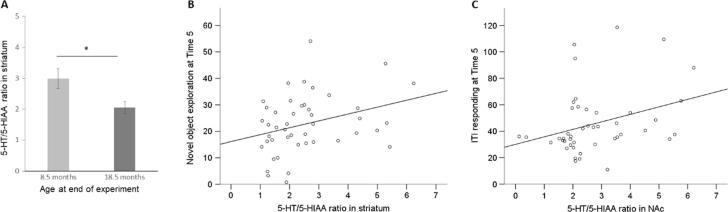
The 5-HT/5-HIAA ratio showing (A) the relative reduction in the dorsal striatum of rats aged 18.5 (as compared with 8.5) months at the end of the experiment, (B) the correlation between the 5-HT/5-HIAA ratio in dorsal striatum and novel object exploration at timepoint 5, and (C) the correlation between the 5-HT/5-HIAA ratio in the NAc and responding in the ITI at the final timepoint. Asterisk shows the significant reduction in the dorsal striatum of the middle-aged cohort (now aged 18.5 months) as compared to the relatively younger group (now aged 8.5 months), *p < 0.05.

Table 1 shows the raw weight corrected tissue levels, for 5-HT, DA and metabolites by brain region and age at the point of sampling. The 5-HIAA/5-HT ratio for dorsal striatum was lower in the middle-aged cohort, t(43) = 2.474, p = 0.017 (Fig. 6A). There were no other significant differences in the ratios used to index turnover, maximum t(43) = 1.657. HVA levels were not reliably determined except in dorsal striatum where [DOPAC + HVA]/DA also showed no significant difference, t(40) = 0.288, p = 0.775, so for consistency the ratio used to index DA turnover was calculated as DOPAC/DA. In addition to the overall reduction seen in older animals, the 5-HIAA/5-HT ratio for dorsal striatum showed some correlation with the summary NOR scores at the final test timepoint. Specifically, there was a positive correlation between the 5-HIAA/5-HT ratio in dorsal striatum and exploration of the novel object, r(45) = 0.318, p = 0.033 (Fig. 6B). There were no other correlations between the summary exploration scores and the ratios used to index DA and 5-HT turnover at the final NOR timepoint, maximum r(45) = 0.243.

**Table 1:** Weight corrected tissue levels (±s.e.m.) for 5-HT, 5-HIAA, DA, DOPAC and HVA by brain region and age. The middle-aged cohort were aged 18.5 months and the younger-adult cohort were aged 8.5 months when the samples were taken. Figures in bold show detectable levels in sample sizes too small for further analysis. To avoid the margin of error in the correction for tissue weight, the statistical analyses were restricted to the ratios used to index 5-HT and DA utilisation, computed for each brain region from the raw levels of metabolite and neurotransmitter.

	*Brain Region/Cohort*					
	Dorsal striatum		NAc		mPFC	
*Levels*	Middle-aged	Younger-adult	Middle-aged	Younger-adult	Middle-aged	Younger-adult
5-HT	1.5319 (±0.3186)	1.0533 (±0.1183)	1.1824 (±0.1577)	1.1150 (±0.1476)	0.7910 (±0.0996)	0.6570 (±0.1042)
	N = 21	N = 24	N = 21	N = 24	N = 20	N = 23
5-HIAA	2.4957 (±0.3287)	2.6700 (±0.2414)	3.2005 (±0.4024)	2.5304 (±0.2279)	2.2980 (±0.2288)	2.1248 (±0.2732)
	N = 21	N = 24	N = 21	N = 24	N = 20	N = 23
DA	15.4052 (±3.7256)	15.8392 (±3.2064)	13.1195 (±1.9865)	10.3863 (±2.0598)	0.3560 (±0.0475)	0.2674 (±0.0499)
	N = 21	N = 24	N = 21	N = 24	N = 20	N = 23
DOPAC	6.5438 (±0.8418)	11.8679 (±2.0145)	15.6695 (±2.1629)	15.2254 (±3.3323)	0.5339 (±0.0927)	0.4900 (±0.0907)
	N = 21	N = 24	N = 21	N = 24	N = 18	N = 23
HVA	2.6637 (±0.3111)	4.1504 (±0.6215)	**3.5986 (±0.7063)**	**3.6711 (±1.0052)**	**0.0650 (±0.0191)**	**0.0140 (±0.0040)**
	N = 19	N = 23	**N = 7**	**N = 7**	**N = 6**	**N = 5**

In relation to the summary TC measures, there was a positive correlation between the 5-HIAA/5-HT ratio in the NAc and responding in the ITI at the final timepoint, r(45) = 3.23, p = 0.031 (Fig. 6C). There were no such correlations between responding in the ISI and the ratio measures (2 s maximum r(21) = 0.346; 10 s maximum r(24) = 0.217); and there were no further correlations between responding in different parts of the TC sessions and the ratios, maximum r(45) = 0.218.

## 4 Discussion

The ages examined in the present study are approximately equivalent to human ages 45–60 years (in the middle aged cohort) versus 20–35 years (in the younger-adult cohort). Thus, the rats in the older cohort should have been pre-senescent, at least at the start of the study. Additionally, age-irrespective, rats were expected to show positive transfer from earlier training sessions which would counteract age-related decline. On these grounds, behavioural changes indicative of cognitive impairment might be expected to be modest. Nonetheless, statistically significant effects involving timepoint and cohort confirmed that there were two age-related differences in performance in the appetitive TC procedure: (1) reduced improvement in conditioning from one day to the next in the middle-aged cohort, at the first timepoint measures of responding to the 2 s trace CS (Fig. 2); and (2) evidence to suggest impaired timing ability in the middle-aged cohort (Fig. 3).

Testing conditioning over successive days and at different timepoints over an extended period requires retention to show improvement (Oswald et al., 2008, Oswald et al., 2010, Runyan et al., 2004). Therefore the reduced improvement from one day to the next in the middle-aged cohort, at the first timepoint measures of responding to the 2 s trace CS, points to impaired retention of prior learning. This difference was not further amplified by aging during the course of the present study but rather reduced over the later timepoints. Over and above any age-related deficit, CS responding increased with repeated testing at the 2 s trace interval in both age groups. Indeed older rats showed a relatively greater increase in CS responding from timepoint 1 to timepoint 5, but from a lower baseline than the younger-adult cohort. In contrast, levels of ITI responding dropped and US responding was maintained in the middle-aged cohort (Fig. 1). Thus – because the pattern of changes over time was different – differences in general activity levels and motivation do not seem to account for the aforementioned changes in CS responding. Moreover the use of a difference score for the analyses (calculated by subtracting the average responding within 5 s of the ITI from the CS responding) provided a control for non-specific effects.

Analyses of responding within 10 s trace ISI bins showed that whilst the younger-adult rats eventually distributed their responding towards the latter part of the ISI, there was no such tendency in the middle-aged cohort; neither was there any evidence that the middle-aged cohort expected the US deliveries any earlier – responding remained evenly distributed across the 2 s bins of the ISI. In the absence of motivational differences between the age-groups (as suggested by the similar levels of US responding), this pattern of findings may reflect some age-related deficit in timing ability. In human eyeblink conditioning, it has been reported that aged participants showed impaired TC but preserved ability to time blink CRs in relation to airpuff US deliveries, but the trace interval is much shorter in eyeblink procedures (500 ms; Cheng et al., 2010).

### 4.1 Comparison with studies of aversive TC

Aversive studies have demonstrated TC impairments in aged animals, e.g. using eyeblink conditioning in rabbits (Graves & Solomon, 1985) and mice (Galvez et al., 2011, Kishimoto et al., 2001), as well as fear conditioning in the rat (McEchron et al., 2004, Moyer and Brown, 2006). These studies also provide evidence that TC deficits can appear as early as middle age (Kishimoto et al., 2001), albeit hearing deficits can be a contributing factor in C57 mice when the CS is auditory (Galvez et al., 2011). Aversive TC procedures have the advantage that only one or two days of conditioning are necessary; however (precisely because of the speed of acquisition) they are not well suited to the use of repeated testing sessions. Thus, aversive TC has typically been employed in cross-sectional rather than longitudinal studies of aging. Appetitive procedures – of the kind adopted in the present study – are more suitable for the repeated testing which is necessary to examine TC longitudinally.

TC, including the fear conditioning variants employed in studies of aging (McEchron et al., 2004, McEchron et al., 2000, Moyer and Brown, 2006, Misane et al., 2005), is known to be dependent on hippocampus (Gabrieli et al., 1995, Misane et al., 2005, Moyer et al., 1990, Solomon et al., 1986, Weitemier and Ryabinin, 2004). However, the demonstration of hippocampal lesion effects in appetitive TC procedures has been challenging (Chan et al., 2014, Tam and Bonardi, 2012, Thibaudeau et al., 2007, Thibaudeau et al., 2009). For example, the length of the ITI relative to the trace interval has been shown to be an important variable for appetitive TC and moreover a determinant of its sensitivity to hippocampal lesion effects (Chan et al., 2014). In the present study the ITI was variable and relatively short, at least 1.5 times longer than the ISI length, as per our previous studies (Pezze et al., 2015a, Pezze et al., 2017, Pezze et al., 2018).

### 4.2 Age-related differences in NOR

It was predicted that the middle-aged cohort of rats should show impaired NOR, particularly at the 24 h retention delay (Burke et al., 2010). It was also expected that, at the second testing session (conducted at timepoint 5), the increased age of both cohorts would result in further NOR impairment. Counter to expectation, further impairment in the ability to discriminate novel and familiar objects was not demonstrated. Just at the first NOR test timepoint, the discrimination ratios showed chance performance in the middle-aged cohort tested at the 24 h retention interval. Discrimination ratios were otherwise above chance, even in the middle-aged cohort tested at the 24 h retention interval at timepoint 5 (at late middle age). Fig. 5 shows that the middle-aged group showed improvement when re-tested at the final timepoint. This effect can be seen to be due to the fact that the middle-aged rats did so poorly with the 24 h retention interval at the first NOR testing session. Nonetheless the message in these data is clear, taken in isolation, cross-sectional comparisons can suggest age-related impairment, yet this may be overcome with repeated testing. This recovery in performance was seen despite the drop in activity levels in older animals, manifest as reduced sample exploration (which could in principle be expected to impair NOR).

Thus, in line with the further acquisition seen in the TC procedure, NOR discrimination was equivalent when the cohorts reached 8 and 18 months of age. The only behavioural parameter which changed over time and was different by age group was sample exploration (Fig. 4). Specifically, the time spent exploring the available objects at the sample stage reduced longitudinally as well as showing a clear difference between age groups, the middle-aged cohort exploring less to begin with. Possibly NOR variants based on object location or recency of presentation would have shown effects of aging also on discriminative accuracy. In mice, normal aging has been found to impair memory for object location (Wimmer, Hernandez, Blackwell, & Abel, 2012). In recency variants, young rats will explore the least recently presented objects, whereas older rats have impaired memory for temporal order and explore objects equally, irrespective of when the objects were seen (Hauser, Tolentino, Pirogovsky, Weston, & Gilbert, 2009).

### 4.3 How were the effects mediated neurochemically?

First, the results showed an overall reduced 5-HIAA/5-HT ratio in the dorsal striatum of the middle-aged cohort. This finding is consistent with the most striking age-related behavioural change which was in activity as measured by NOR sample exploration (Fig. 4). Second, there were also some correlations between the ratios used to index neurotransmitter turnover in the striatum and behavioural parameters. For NOR, there was a positive correlation between the 5-HIAA/5-HT ratio in dorsal striatum and test exploration of the novel object at the final timepoint. For TC, there were positive correlations between the 5-HIAA/5-HT ratio in the NAc and responding in the ITI at the final timepoint (Fig. 6).

Thus, changes in 5-HT function were linked to activity-related parameters in both TC and NOR procedures. Reduced striatal 5-HT turnover might account for overall decreased object exploration but was nonetheless unrelated to NOR cognitive performance. It is well-established that 5-HT modulates motor function and behavioural arousal (Jacobs & Fornal, 1997) and both 5-HT and DA influence the timing of responding, as measured in peak (Balci et al., 2008, Morrissey et al., 1994) and interval timing procedures (Meck et al., 2012). However, in the present study there were no correlations between neurochemical parameters and responding in the ISI of the TC procedure.

### 4.4 Further limitations of the design

Due to circumstances beyond our control, there was a confound in the design in that we had to order the younger cohort of Wistar Han rats from a different barrier. This switch in supply could have compromised comparison between the groups if there were systematic differences in behavioural phenotype between the barriers. Goepfrich, Gluch, Friemel, and Schneider (2013) compared Wistar lines (HsdHan vs RccHan) derived from the same breeder (Harlan) as well as differences between breeders (Harlan vs Janvier). They found that the Wistar rat line made more difference than the supplier. In the present study, both cohorts of rats were from the same line and supplier: Wistar RccHan (ordered through the same contact at Harlan Bicester UK and confirmed to be of matched genetic background and health status).

It must also be acknowledged that the design did not include further groups of untrained aged rats, for inclusion in the neurochemical analyses, to examine the effects of age independent of training. However, animals do not age in a vacuum and whatever the experience of these animals it would doubtless beg further questions as to the role of environmental enrichment and other aspects of the husbandry regime in healthy brain ageing. Thus there is a more general limitation to be considered in that the time rats take to age inevitably conflates their experiences to that point: environmental enrichment and social caging (for example) would be expected to mitigate against the effects of aging. The younger rats were delivered at 6 weeks of age, and (as is standard in all our behavioural studies) acclimatized in our animal unit for two weeks before the behavioural testing was started. The older rats were delivered at 16 weeks of age and further aged in house until they reached 12 months. Thus the older cohort experienced an additional 10 weeks under the suppliers’ conditions and some 8 months in our animal unit. In house aging was preferable in that we could achieve some weight control during the aging period (following the standard 2-week acclimatization) and the regular handling to weigh them (every 2–3 days) reduced the rats’ reactivity. The provision of cardboard tubing, chew sticks and nesting materials is standard in our animal unit. We have no way to show what the effect of husbandry on in house aging may have been but our presumption is that our handling and enrichment regime should have (if anything) reduced the cognitive deficits otherwise associated with aging, possibly for non-specific reasons related to reduced reactivity in novel situations and/or the health benefits of the food restriction. In house aging guaranteed the availability of the older group of rats at the time scheduled to complete the study and – although the environment was not enriched beyond our standard level – we could at least be confident that any age-related impairment was not simply attributable to environmental deprivation rather than older age per se.

Possibly related to the use of environmental enrichment during the in house aging (while the middle-aged cohort reached 12 months and during the longitudinal component of the design for both cohorts), the age-related impairments shown in the present study were not particularly marked. The adaptation of the appetitive TC procedure for the longitudinal aspect of the design may also have been suboptimal. For example, there were no interim extinctions sessions with the noise CS. These would not have eliminated the effects of earlier conditioning sessions but they would be expected to reduce the positive transfer of learning from one timepoint to the next. However, any age-related differences in the course of interim extinctions would have confounded the assessment of acquisition at the subsequent timepoints. In any case, based on previous work with the same conditioning procedure there were no grounds to assume that conditioning would be at asymptote after in total 60 conditioning trials at timepoint 1 (Cassaday et al., 2005, Pezze et al., 2015a, Pezze et al., 2017, Pezze et al., 2018). Indeed, at the 2 s trace interval, 2 s TC continued to improve for both age-groups in the present study. Thus ceiling effects should not have precluded seeing differences between groups or longitudinally as the rats continued to age. However, the results showed only that there was reduced improvement from one day to the next in the middle-aged cohort, at the first timepoint measures of responding to the CS in the 2 s trace interval group. The age-related deficit in the 10 s trace group seemed to relate to a timing deficit, taking the form that – different from the profile of responding of the younger-adult group – the middle-aged cohort did not learn to respond later in the trace interval.

There was no evidence at all for any age-related differences in contextual conditioning, as measured by responding to the experimental light background. However, conditioning to the light background was directly assessed only by a single extinction test 2 weeks after the end of an extended training (consisting of in total 10 days of 30 trials, conducted over 5 two-day time points spanning the 6 month longitudinal study). Possibly, stronger evidence of conditioning to the light background would have been obtained had this been tested at an earlier timepoint. A further consideration arises in that age-related differences in the sensitivity to distracting stimuli could have confounded interpretation of the rats’ responding to the flashing light background stimulus or the target CS. Distractors have been found to interfere with trace conditioning (Han et al., 2003; but see Pezze, Marshall, & Cassaday, 2016). However, as trace conditioning showed improvement with repeated testing we would have to assume that distractibility decreased with age. A better measure of contextual conditioning might have been provided by measuring responding in the context, prior to the first CS-US trial on each training day, but this interval was variable rather than fixed in the procedure we have been using (Pezze et al., 2015a, Pezze et al., 2017, Pezze et al., 2018).

### 4.5 Conclusions and implications

Counter to expectation, age-related decline was not demonstrated longitudinally as rats progressed through to middle age. On the contrary, the results showed further acquisition at the 2 s trace interval at the later conditioning timepoints. There was, however, evidence for reduced improvement from one day to the next in the middle-aged cohort, at the first timepoint measures of responding to the 2 s trace conditioned stimulus. Neither cohort showed much responding to the CS when this was conditioned at the 10 s trace interval, but there was evidence for impaired timing within the 10 s trace in the middle-aged cohort. This difference between the cohorts increased over the course of the study not because of any further impairment with further aging but because timed responding developed only over the extended period of training in the younger-adult cohort.

Notwithstanding the challenges posed by designs of the kind used in the present study, the results underscore the importance of longitudinal studies. Between groups comparison showed NOR discriminative impairment in middle-aged rats tested at the first timepoint (at a 24 h but not at a 10 min retention interval). Object exploration at the sample stage of the procedure was overall reduced in middle-aged rats and further reduced longitudinally. However, despite further reduced sampling of the objects, the middle-aged rats showed recovery when re-tested on NOR at the final timepoint. The difference in 2 s TC between the middle-aged and younger-adult cohorts was similarly transient. Thus taken in isolation, cross-sectional studies may overestimate age-related impairments.

Age-related decline in middle-age might be expected to be modest and potentially difficult to demonstrate over the course of an extended acquisition with the appetitive TC procedure in use. Nonetheless early behavioural changes are key to the detection of biomarkers for the onset of age-related cognitive decline, prior to the onset of irreversible neuropathological changes, offering prospective screening tools to assess the efficacy of interventions to restore behaviour, for example with DA D1 (Pezze, Marshall, & Cassaday, 2015) or muscarinic receptor agents (Pezze et al., 2017, Pezze et al., 2018) tested in older animals undergoing TC.

TC is likely supported by a wider network of distributed mechanisms (Gilmartin et al., 2014) and, of the areas sampled in the present study, only dorsal striatum showed any overall difference between the cohorts: specifically, the 5-HIAA/5-HT ratio was lower in the middle-aged group. Moreover, the 5-HIAA/5-HT ratios in the striatal sub-regions were the only measure to show any correlation with the behavioural parameters and notably those correlations were with measures related to exploratory activity. Thus, the findings of the present study also suggest that measures of striatal function may provide a useful proxy for the risk of cognitive decline as rodents age. Measures of activity and general exploration were lower in older animals and showed further decline with aging. These changes (shown in the ITI of the TC procedure and at the sampling stage of the NOR procedure) also show the importance of taking non-specific effects into account in studies of aging rodents, particularly given that the vast majority of learning and memory tasks involve some degree of movement or its suppression (Izquierdo et al., 2006).

## Author contributions

All of the authors have made a substantial contribution to the experimental design, data analyses and/or write up of the study. The submitted version of the manuscript has been seen and approved by all authors and none of the data have been previously published (except in abstract form for conferences). This work was supported by the BBSRC (ref. BB/K004980/1). The authors have no competing financial interests or other conflicts of interest and nothing to disclose. The BBSRC had no further role in the design of the study, collection and analysis of data or the decision to publish the data.
